# An Odontome

**Published:** 1880-03

**Authors:** G. V. Black


### ARTICLE IV.

### *An Odontome.*

BY G. V. BLACK.

I have here an erotic formation, to which I wish to call the attention of the members of this Society for a few moments only. It is an Odontome, Odontomata or tooth tumor, or a tooth the formation of which is so faulty that it has not the appearance of a tooth. This specimen occurred in the practice of Dr. E. Duncan, of Jacksonville. The patient a man about twenty-five years old. It occupied the position of the second molar in the upper jay, and had never given any serious trouble; but in the last few months had become loosened, and at last dropped out without any scientific aid whatever. Dr. Duncan had examined it a short time before this, while the Odontome was in position, and again examined the mouth a few days after it had dropped out, and found the wisdom tooth, which had been missing, making its appearance in the socket left by the Odontome. It is evident that the wisdom tooth had been detained by being beneath the Odontome. The other wisdom teeth were all in their places, as were all the other teeth; they were well formed and free from decay. The health of the patient, and that of his family, had been good, and the teeth of all well formed.

When dry, the weight of the Odontome was 4.77 grains. Measuring according to the situation in the mouth, it measures

24 millimeters from before backward,
20 " from side to side,
14 " from root to crown.

The upper or crown surface is oval, presenting no sharp angles, but is somewhat irregular in form; yet showing no appearance of normal cusps. From before backwards it is nearly flat from side to side; portions are quite smooth and covered with enamel, other portions rough and apparently without enamel. This upper surface or crown portion overhangs the lower or root portion a little all round the margin, and in most parts presents the appearance of an irregular junction of enamel with dentine.

The lower or root portion is generally flat, though quite irregular, presenting a number of depressions and elevations—notably one large depression in the centre of the long diameter and the posterior half of the short diameter, which, from its form, would seem to have been moulded upon a prominent cusp of the coming wisdom tooth, upon which it seemed to have fitted. Around this depression there were irregular protuberances, from two to four millimeters in height. The anterior third of this surface slopes up to the junction of the enamel, while the remaining portions, although quite irregular, rise pretty squarely to the junction with the enamel.

For the purpose of examining its structure, I have sawed it through, halving it in an antero-posterior perpendicular direction, and cut some sections. I found all the tissues of a normally developed tooth, but in a state of confusion. There is entire absence of any proper pulp cavity.

The disposition or arrangement of the tissues is very peculiar and striking. It is as though there were a thousand little teeth, exceedingly minute, growing as close together as they could be crowded, and the interstices between them filled up with enamel and cement. In the field of the microscope, with the sections I have, we will often be able to see a number of these diminutive teeth at a single view; each has its own little pulp chamber in due form, its own separate dentine, and its own enamel cap, and plastered in and about, and added on to these, there is a considerable amount of both enamel and cement of very irregular formation. Very many of the pulp chambers are partially filled with calcosphereites. These also appear in many parts of the specimen in profusion.

It is interesting to note the resemblance of this Odontome to the normal structure of the teeth of some of the lower orders of animals, especially some species of fishes, in which there are branching and radiating pulp cavities. These forms are well described by Charles Tomes.

These forms of Odontomata are exceedingly rare, if we may judge from the reports we have thus far been able to find; yet it is important that they be properly understood when they do occur, for they do not always drop out of themselves as this one has done, but are inclined to give very serious trouble. We will give a condensed resume of the cases reported thus far; that is, all that have come under our notice. In doing this, we exclude everything except the complete malformation of both root and crown. They seem to constitute a distinct type, and are more properly *dental tumors* than malformed teeth, and therefore merit the name of Odontomata or Odontome, which we do not think should be applied to simple misshapes of the teeth.

Case 1. Reported by Dr. Jarish, etc. (from Wedl)—Patient, female, 25 years old, weak of body. A tumor was noticed on the back part of jaw. Inflammation occurred, with formation of abscess. A malformed tooth was removed with forceps.

Case 2. Reported by Prof. Strasky, of Lundberg—Patient 20 years old. Presented tumor on right cheek of several weeks' duration; difficult deglutition and much pain; the jaws were opened with difficulty sufficiently to make an examination. Gums found inflamed and swollen. Diagnosis: Impeded wisdom tooth. Gum was dissected up, and an irregular mass removed with forceps. The wisdom tooth was found directly under it.

Case 3. Reported by Dr. Steinberger—Patient, female, aged 15 years. Presented very great swelling of the face, with formation of abscess at the angle of jaw. Results same as others.

Case 4. Reported by Mr. Harrison as occurring in pauper lunatic in an asylum in London—This, after much sloughing of the parts, came away of itself. It was situated in the region of the bicuspids, and was different from the others in its histological character.

Case 5. Reported by J. Tomes—An incorrect diagnosis made, and a section of the lower jaw removed. An examination made after removal showed it to be an Odontome exactly similar to the one here shown.

Case 6. Reported by Wedl as occurring in the practice of a distinguished surgeon—A wrong diagnosis was made, and a section of the lower jaw excised.

Case 7. Reported by M. Forget, of Paris—Patient, male, age 20. His sufferings began many years before. All the permanent molars on the right side of the lower jaw were wanting, having never made their appearance, and their place was occupied by a large, hard tumor. The soft parts were swollen and indurated. Pus escaped from several fistulous openings. A large section of the lower jaw was removed. After examination revealed the fact that the trouble was caused by a large Odontome, under which was found the second bicuspid and one of the molars.

These are all the cases of this nature that have come under my notice. All of them have been similar to the one I have shown, as to the structure of the malformed mass, except the one reported by Mr. Harrison, which presented a distinct pulp chamber and a more regular formation of the tissues, though, as a tooth, it was wholly misshapen.

These, it seems to me, stand out very distinctly as a class of Odontomata, tooth tumors, or Odontomes, and should not be confounded with mere excrescences upon otherwise well-formed teeth, or malformation of some particular part of a tooth otherwise well-formed. What may be the causes leading to these erratic growths I do not know, but there can be no doubt that they arise from some perversion of the tooth germ. It is quite remarkable that in five out of the seven cases, they have evidently sprung from the germ of the second permanent molar, and that in all of these five cases the wisdom tooth was found directly under the mass; and, furthermore, the trouble arising from them has, in every case, been at, or a little after, the usual time that the wisdom tooth should make its appearance. We have therefore, good reason for concluding that in these cases the trouble experienced has been really in consequence of the impediment offered to the coming wisdom tooth by the presence of the deformed mass; while in the case I have now reported, the attachment was so slight that it was pushed out before the coming wisdom tooth.

In the case reported by M. Forget, of Paris, the conditions were somewhat different, though the report we have is not quite clear. However, it seems likely that in this case the germ of the first molar was at fault, possibly including that of the second.

In the case reported by Mr. Harrison, of London, we have more nearly the normal tooth structure, and a situation in the region of the bicuspids. This case is not quite similar to the others.

The fact that in three of these cases a section of the lower jaw has been needlessly removed through error of diagnosis, and the patients been thus horribly maimed for life, throws a dark stain over the history which should stimulate us, as dentists, to be ready to form correct conclusions should such cases be presented to us.—*Trans. Ill. State Dental Society.*

